# Effective passivation of Ag nanowire network by transparent tetrahedral amorphous carbon film for flexible and transparent thin film heaters

**DOI:** 10.1038/s41598-018-31927-z

**Published:** 2018-09-10

**Authors:** Hae-Jun Seok, Jong-Kuk Kim, Han-Ki Kim

**Affiliations:** 1 School of Advanced Materials Science and Engineering, Sungkyunkwan University, 2066, Seobu-ro, Jangan-gu, Suwon-si, Gyeonggi-do, 16419 Republic of Korea; 2 Department of Surface Process Laboratory, Korea Institute of Materials Science, 797, Changwon-aero, Seongsan-gu, Changwon-si, Gyeongsangnam-do, 51508 Republic of Korea

## Abstract

We developed effective passivation method of flexible Ag nanowire (NW) network electrodes using transparent tetrahedral amorphous carbon (ta-C) film prepared by filtered cathode vacuum arc (FCVA) coating. Even at room temperature process of FCVA, the ta-C passivation layer effectively protect Ag NW network electrode and improved the ambient stability of Ag NW network without change of sheet resistance of Ag NW network. In addition, ta-C coated Ag NW electrode showed identical critical inner and outer bending radius to bare Ag NW due to the thin thickness of ta-C passivation layer. The time-temperature profiles demonstrate that the performance of the transparent and flexible thin film heater (TFH) with the ta-C/Ag NW network is better than that of a TFH with Ag NW electrodes due to thermal stability of FCVA grown ta-C layer. In addition, the transparent and flexible TFHs with ta-C/Ag NW showed robustness against external force due to its high hardness and wear resistance. This indicates that the FCVA coated ta-C is promising passivation and protective layer for chemically weak Ag NW network electrodes against sulfur in ambient.

## Introduction

Ag nanowire (NW) percolating network electrodes coated on flexible substrate has been extensively investigated as a promising replacement of high-cost and brittle indium tin oxide (ITO) films in flexible optoelectronics due to its solution-based simple printing process, low-cost ambient coating process, low resistivity, and high transmittance^1–4^. In particular, outstanding flexibility or stretchability of Ag NW electrode against substrate bending or stretching is one of key merits of the Ag NW network electrodes^5–9^. For those reasons, simply printed Ag NW network electrode have showed a potential as transparent and flexible electrodes in flexible displays, flexible touch screen panels, flexible photovoltaics, flexible sensors and flexible thin film heater (TFHs)^10–20^. In spite of attractive merits, the Ag NW films have critical drawbacks such as poor adhesion with flexible substrate, non-uniform topography, easy degradation under ambient conditions, and instability against static electricity. In particular, as discussed by Elechiguerra *et al*., Ag NW prepared by polyol method was sulidized by reduced-sulfur-containing gas (H2S) at ambient conditions^21^. In addition, the current constriction on Ag NWs led to the local melting of the Ag NWs or degradation of the flexible substrate during the Joule heating process of thin film heaters. To prevent sulfidation of Ag NWs in the atmosphere, organic or inorganic of passivation layers such as graphene, conducting oxide, conducting polymer, carbon nanotube and graphene oxide has been employed as transparent passivation layers^22–30^. Those transparent passivation layer improved connectivity of Ag NW and protected the Ag NW from reduced-sulfur-containing gas in ambient. Although several passivation layer have been studied, there are no reports on a tetrahedral amorphous carbon (ta-C) layer or hydrogen free carbon prepared by filtered cathode vacuum arc (FCVA) coating as a transparent and flexible passivation layer for the Ag NWs network electrodes. To obtain amorphous carbon (a-C) films with high degree of sp^3^ bonding, several deposition techniques have been suggested^31,32^. In particular, Mckenzie *et al*., denoted a-C as ta-C to distinguish form sp^2^ a-C. These ta-C films have been grown using a wide variety of process including filtered cathodic vacuum arc (FCVA)-direct and pulsed source, pulsed laser ablation, mass selected ion beam deposition, and electron cyclotron wave resonance processes^31–35^. Among those techniques, the FCVA coated ta-C films has been known as an excellent coating materials for mechanical parts due to their interesting properties caused by high sp^3^ contents such as low friction coefficient (<0.1–0.25), high hardness (80 GPa), optical transparency, moderate conductance, atomically smooth surface and chemical stability^36–50^. Those attractive mechanical properties of the ta-C film is well apt for the passivation and protective layer of Ag NW network electrode in transparent and flexible TFHs.

In this work, we report on highly transparent and mechanical flexible ta-C passivation layer for Ag NW network prepared by FCVA method at room temperature. The FCVA coated ta-C layer on Ag NW network act as an effective passivation layer against sulfidation and improved Ag NW-based TFHs. In particular, we investigated the effect of ta-C thickness on the electrical, optical and mechanical properties of Ag NW network electrodes to optimize the passivation thickness. Furthermore, we compared the performance and mechanical stability of TFHs with bare Ag NW and ta-C coated Ag NW electrodes to demonstrate potential of the FCVA coated ta-C passivation layer for high performance transparent and flexible TFHs.

## Results

**Figure 1:**
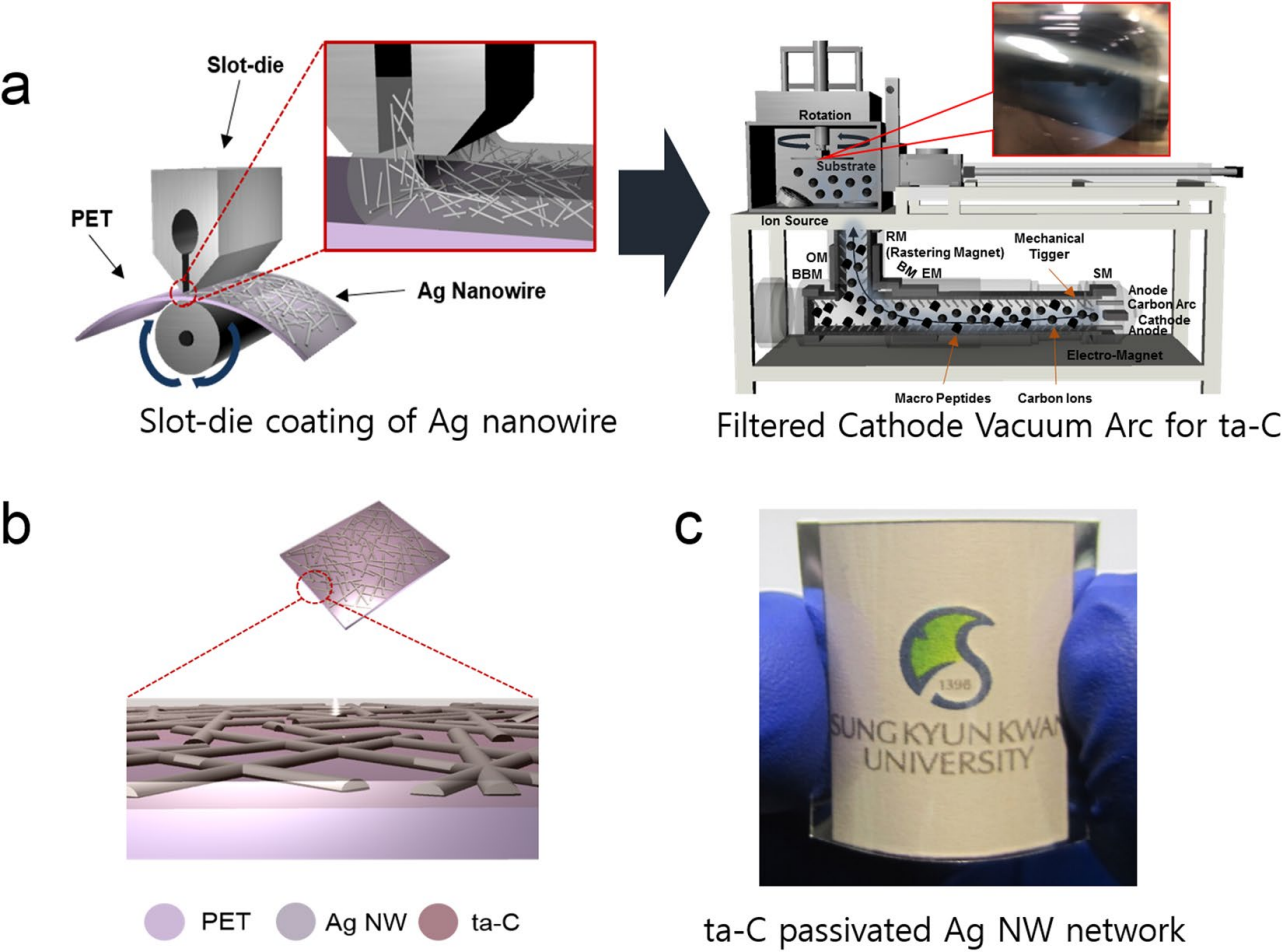
(**a**) Schematics of slot-die coating of Ag nanowire network on 700 mm width PET substrate at room temperature and FCVA system for coating of ta-C passivation layer on the Ag NW network. Picture in FCVA system showed arc plasma beam. (**b**) Schematics of ta-C passivated Ag NW network electrode on PET substrate. (**c**) Transparent and flexible thin film heaters fabricated with ta-C coated Ag NW electrode.

Figure 1a showed schematic fabricatrion process of flexible and transparent Ag NW network electrodes passivated by a thin ta-C layer. Using commercial Ag NW ink and 700 mm wide roll to roll slot-die coater, we fabricated transparent and flexible Ag NW network electrode on PET substrate as illustrated in left side of Fig. 1a. Then, ta-C passivation layer was directly coated on the slot-die coated Ag NW network electrode using lab-scale FCVA system. During FCVA coating process, a plasma beam with carbon-related macro-particles and neutral carbon is emitted from the cathodic arc spot on the carbon target. Photograph in Fig. 1a shows the arc plasma beam moving through the filter from the cathodic arc source. Figure 1b showed the schematic of ta-C coated Ag NW network electrode structure for transparent and flexible TFHs. Figure 1c demonstrated transparent and flexible TFH fabricated on ta-C passivated Ag NW electrode.

**Figure 2:**
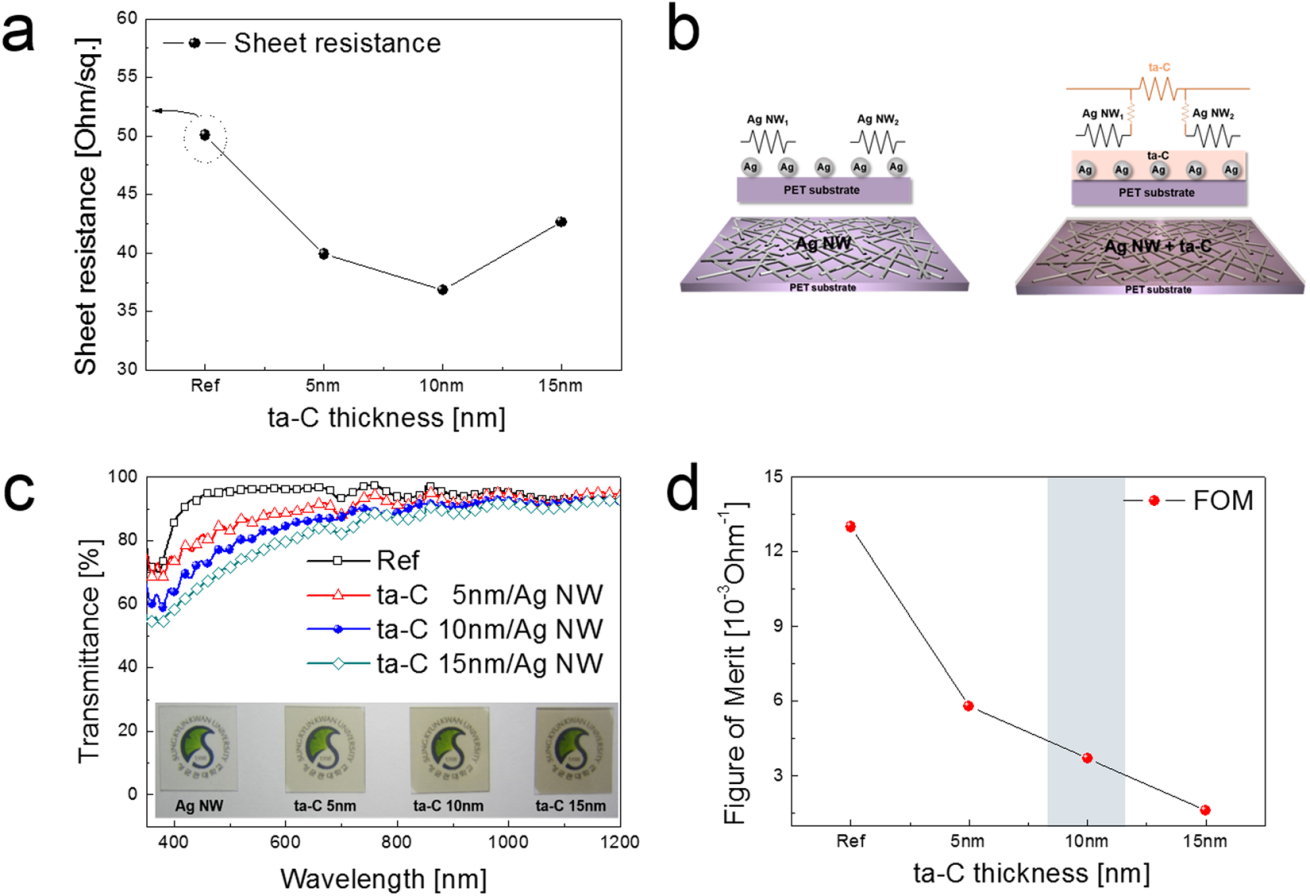
(**a**) Sheet resistance of ta-C coated Ag NW network electrodes as a function of ta-C thickness. (**b**) Comparison of equivalent resistance circuit diagram for bare Ag NW and ta-C/Ag NW network electrodes. (**c**) Optical transmittance of ta-C/Ag NW/PET samples with different ta-C passivation thickness. Inset pictures showed the color and transparency of the ta-C coated Ag NW electrodes. (**d**) Calculated figure of merit (FOM) values obtained from sheet resistance (Rsh) and optical transmittance (T) of the ta-C/Ag NW films with increasing ta-C thickness.

Figure 2a shows the sheet resistance of the Ag NW network electrodes passivated by FCVA coated ta-C layer with increasing thickness from 5 to 15 nm. As discussed in our previous works, the saturation voltage and temperature of TFHs were critically dependent on the sheet resistance of transparent electrodes^5^. Therefore, to obtain higher performance of transparent and flexible TFHs, low sheet resistance of transparent electrode is very important parameter. Compared to the bare Ag NW electrode with a sheet resistance of 40.51 Ohm/square, the ta-C coated Ag NW electrode showed decreased sheet resistance. Slightly reduced sheet resistance of ta-C coated Ag NW could be attributed to bridge effect of moderate conductive ta-C layer^51^. The 10 nm thick ta-C coated Ag NW network electrode showed the lowest sheet resistance of 36.81 Ohm/square. However, further increase in thickness of ta-C passivation layer led to increase of sheet resistance. In general, electrical properties of carbon-based materials such as graphite, diamond, amorphous carbon (a-C), nanotubes, fullerenes (C_60_), graphene, and ta-C largely affected by the hybridization state and ordering of the carbon-carbon bonds (i.e., sp, sp^2^, and sp^3^)^52–54^. In case of the FCVA coated ta-C films, it was reported that high sp^3^ content (~80%) in the ta-C films resulted in unique mechanical, optical and electrical properties^55^. Undoped ta-C films have been shown p-type semiconducting properties and an optical bandgap 1–2.5 eV ranges^43^. Therefore, nano-scale thick ta-C films on Ag NW could act as bridge between Ag NWs and showed fairly high optical transmittance. Figure 2b shows equivalent resistance circuit diagram of bare Ag NW and ta-C/Ag NW electrodes, respectively. The higher sheet resistance of the bare Ag NW electrode can be understood using the equivalent resistance circuit diagram. In the bare Ag NW network electrode without ta-C passivation, some Ag NWs were only weakly connected or were disconnected, therefore, the bare Ag NW electrode showed a fairly higher sheet resistance due to existence of disconnected Ag NWs. However, the FCVA coated ta-C/Ag NW electrodes showed a decreased sheet resistance due to the bridge effect of the transparent and semiconducting ta-C passivation layer between disconnected Ag NWs as shown in Fig. 2b. Grierson *et al*., reported that ta-C film has moderate conductivity due the existence of trigonally bonded carbons^53^. Chen *et al*., also reported that the FCVA grown ta-c layer on ITO anode act as hole transport layer in organic light emitting diodes due to its p-type conductivity^56^. Fig. 2c shows the dependence of optical transmittance of the ta-C/Ag NW electrodes on the thickness of ta-C thickness at wavelength region between 400 and 1200 nm. Bare Ag NW electrode showed an optical transmittance of 95.81% at a wavelength of 550 nm. However, the ta-C/Ag NW electrode showed slightly lower optical transmittance specially in visible wavelength region between 400 and 800 nm than bare Ag NW electrode due to absorption in the ta-C passivation layer. At the 15 nm thickness of ta-C layer, the ta-C/Ag NW electrodes showed optical transmittance of 76.14%. Therefore, to apply ta-C film as transparent passivation layer, it is desirable to optimize the thickness below 15 nm. The inset pictures demonstrated color and transparency of the bare Ag NW and ta-C/Ag NW electrodes with different ta-C thickness. Compared to bare Ag NW electrode, the ta-C coated Ag NW showed gray color. However, those transparency of the ta-C/Ag NW network electrode is acceptable to fabricate transparent and flexible TFHs. Figure 2d showed figure of merit (FOM) values of the ta-C/Ag NW network electrode with increasing ta-C thickness. The FOM (=T^10^/R_sh_) was calculated from sheet resistance (R_sh_) and optical transmittance (T) of electrode at 550 nm wavelength^57^. The FOM of the ta-C coated Ag NW network electrodes are less than FOM value (13 × 10^−3^ Ohm^−1^) of the bare Ag NW network electrode due to the decreased optical transmittance. Therefore, we determined the optimal ta-C thickness as 10 nm to fabricate transparent and flexible TFHs. At optimized thickness of the ta-C passivation layer, the ta-C/Ag NW/PET sample showed a sheet resistance of 36.83 Ohm/square and optical transmittance of 81.99%.

**Figure 3:**
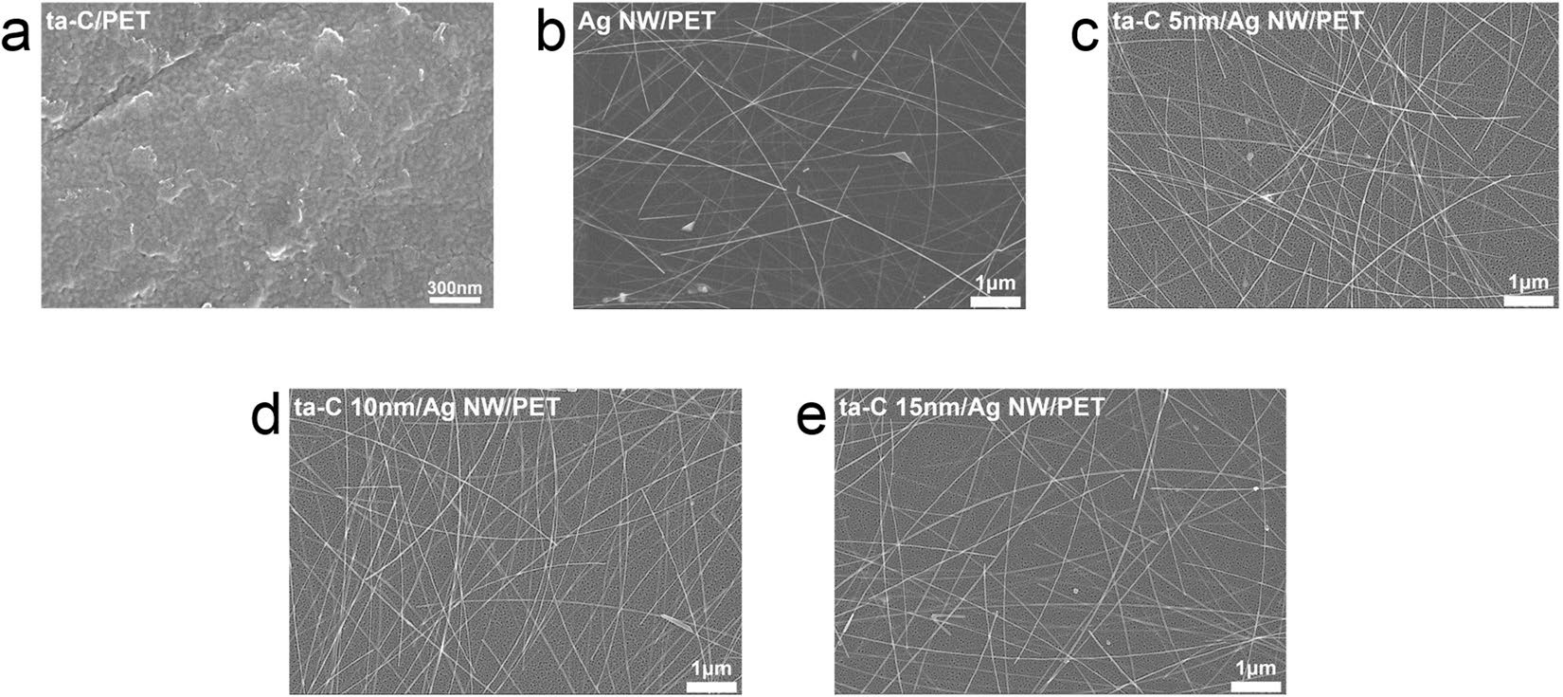
Surface FESEM images obtained from (**a**) bare ta-C (10 nm)/PET, (**b**) bare Ag NW/PET (**c**) FCVA coated ta-C (5 nm)/Ag NW/PET (**d**) FCVA coated ta-C (10 mm)/Ag NW/PET, and (**e**) FCVA coated ta-C (15 nm)/Ag NW/PET.

Figure 3 shows surface FESEM images of bare ta-C, bare Ag NW and ta-C coated Ag NW network electrodes prepared on PET substrates with increasing ta-C layer thickness. The surface FESEM image of the bare ta-C film showed a typical amorphous surface morphology without surface defects such as pin holes, cracks, and protrusions in Fig. 3a. Due to low process temperature of FCVA, the ta-C film exhibited a featureless surface morphology. It was noteworthy that the ta-C coating on the Ag NW network did not affect on the surface morphology and connectivity of the Ag NW network. Due to very smooth surface morphology and conformable coating of the FCVA coated ta-C layer, the surface morphology of the ta-C coated Ag NW network electrodes Fig. 3c–e is identical to that of bare Ag NW network in Fig. 3b. It was clearly shown that Ag NW network was maintained without disconnecting or melting of junction region even after coating of ta-C layer due to low carbon ion energy during FCVA process. This fully covered ta-C layer on the Ag NW network could act as anticorrosion layer against sulfidation and protection layer against external force simultaneously during operation of Ag NW-based TFHs.

**Figure 4:**
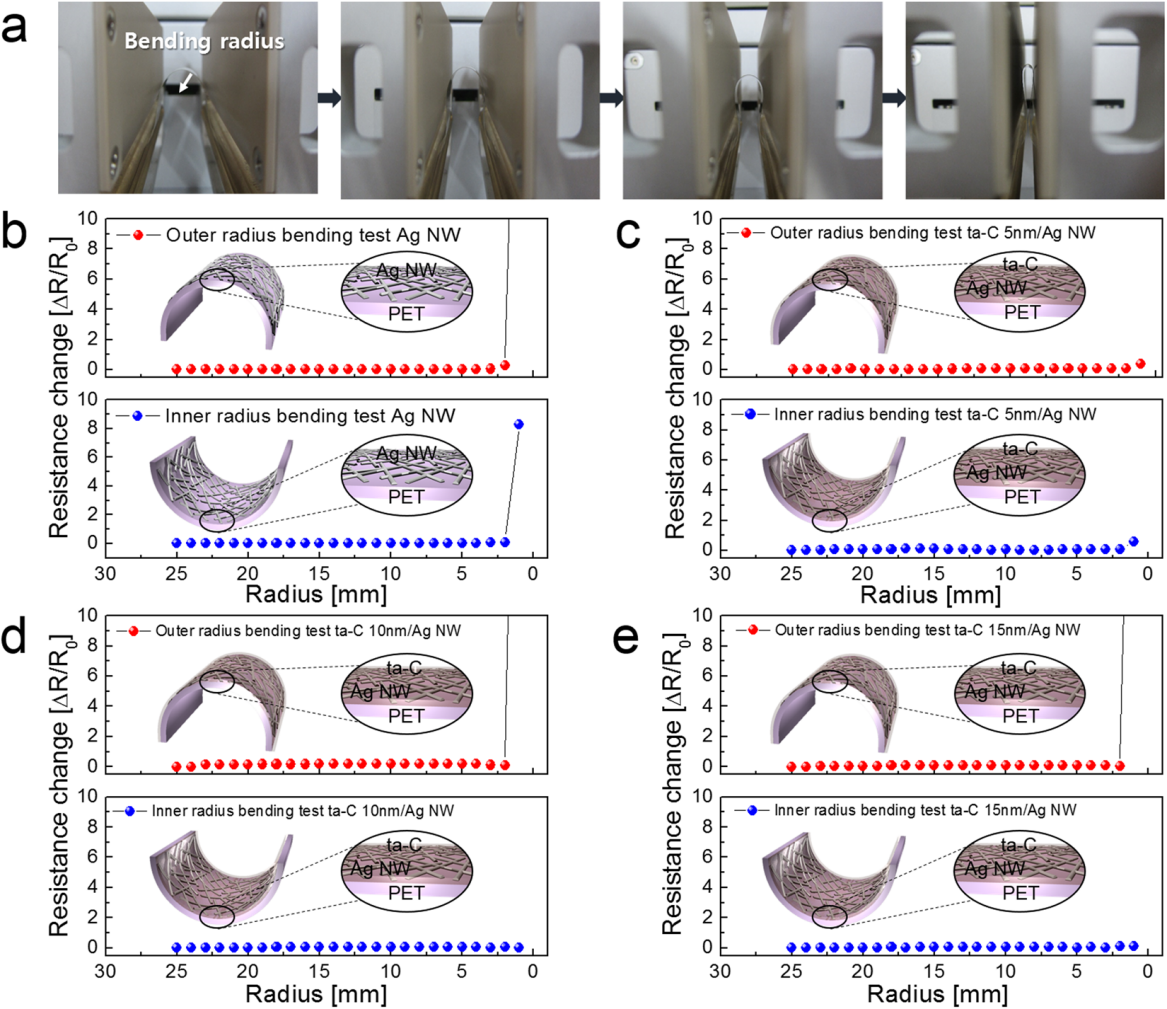
(**a**) Bending steps of flexible ta-C/Ag NW electrode in lab-bade bending test system with decreasing bending radius. Resistance change of (**b**) bare Ag NW, (**c**) ta-C (5 nm)/Ag NWs, (**d**) ta-C (10 nm)/Ag NW, and (**e**) ta-C (15 nm)/Ag NW electrodes with decreasing outer and inner bending radius. Insets show the schematics of curved sample experiencing tensile and compressive stress.

To investigate the feasibility of FCVA coated ta-C film as flexible passivation layer, we measured resistance change of ta-C coated Ag NW electrodes with decreasing bending radius. Figure 4 shows comparison of mechanical flexibility of bare Ag NW and ta-C coated Ag NW network electrode with different thickness of the ta-C layer. Figure 4a showed the pictures of bending steps with decreasing bending radius in lab-made bending test system. The change in *in-situ* measured resistance of clipped samples can be expressed as (ΔR = R − R_0_)/R_0_, where R_0_ is the initial measured resistance, and R is the *in-situ* measured resistance under outer and inner bending. By pressing both side of the clipped sample, we can adjust the bending radius as indicated by arrow. Figure 4b shows outer and inner bending test results with decreasing bending radius from 25 mm to 1 mm. The bare Ag NW network electrode showed a constant resistance change critical outer and inner bending radii, both 2 mm due to outstanding flexibility of Ag NWs network^58–61^. Fig. 4c–e shows outer and inner bending test results of the ta-C coated Ag NW electrodes with decreasing bending radius which is identical bending condition to the bare Ag NW electrode. In spite of coating of thin ta-C passivation layer, the ta-C/Ag NW electrodes showed identical or better flexibility to the bare Ag NW electrode due to very thin thickness of the ta-C layer and conformal coating on the Ag NWs. Below critical outer and inner bending radius, all ta-C/Ag NW electrodes regardless of ta-C layer thickness showed increased resistance change due to disconnection of the Ag NWs in network. Therefore, it was found that the passivation of the transparent ta-C layer on Ag NW did not change of mechanical flexibility of the Ag NW network and could be employed as flexible anticorrosion and protection layer on Ag NW network.

**Figure 5:**
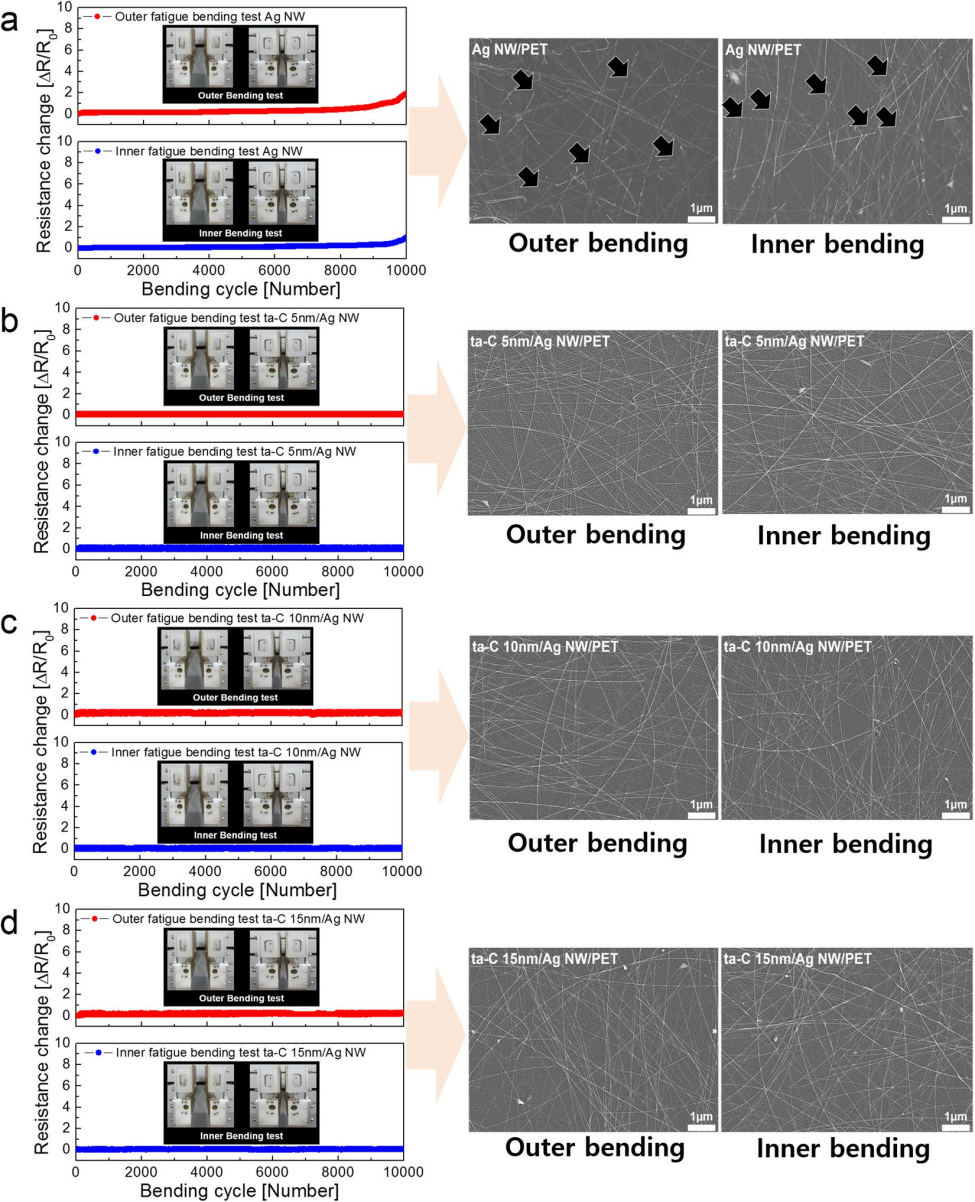
Dynamic outer and inner fatigue tests at a fixed bending radius of 3 mm for (**a**) Ag NW/PET, (**b**) ta-C (5 nm)/Ag NW/PET, (**c**) ta-C (10 nm)/Ag NW/PET, and (**d**) ta-C (15 nm)/Ag NW/PET samples. Inset pictures showed the outer and inner bending steps during dynamic bending test. In addition, surface FESEM images showed morphology and connectivity change after 10,000 cycles.

To investigate stability of ta-C/Ag NW network electrode against repeated bending cycles, we carried out dynamic fatigue test of bare Ag NW and ta-C coated Ag NW electrodes at a constant bending radius of 3 mm as shown in Fig. 5. The bending radius of 3 mm is acceptable value in highly flexible TFHs, which attached on curved surface or used as itself. Inset pictures in left panels shows the outer and inner bending steps during dynamic bending test. Figure 5a showed dynamic outer and inner bending test results of bare Ag NW network electrode as a function of bending cycles. Until 9,000 repeated cycles, there is no resistance change, indicating that maintaining of the Ag NW percolating network. However, above 9,000 cycles, the resistance of Ag NW electrodes showed slightly increased due to disconnection of Ag NWs or delamination of the Ag NWs from PET substrate. The surface FESEM images right side in Fig. 5a obtained from bare Ag NW electrode after 10,000 bending cycles showed the disconnected Ag NW as indicated by black arrows. In particular, the bare Ag NW electrode showed a larger resistance change under repeated tensile stress than compressive stress because tensile stress easily led to disconnection of the Ag NWs^62,63^. However, both dynamic outer and inner bending fatigue tests of the ta-C/Ag NW samples showed no change in resistance even after 10,000 bending cycles regardless of ta-C thickness as shown in Fig. 5b–d. Compared to bare Ag NWs, the ta-C coated Ag NW network electrode has better stability against for repeated bending cycles. Because the conformal coating of ta-C layer on the Ag NW could enhance adhesion of Ag NW on PET substrate, the ta-C coated Ag NW network electrode showed constant resistance change even after 10,000 times repeated bending cycles regardless of the ta-C thickness. The surface FESEM images obtained from the ta-C/Ag NW electrodes after 10,000 outer and inner bending cycles showed identical surface morphology to pristine sample. This outstanding stability of ta-C coated Ag NW can be attributed to the effect of ta-C passivation layer, which enhance the adhesion of the Ag NW network and reinforced the Ag NW against repeated external force.

**Figure 6:**
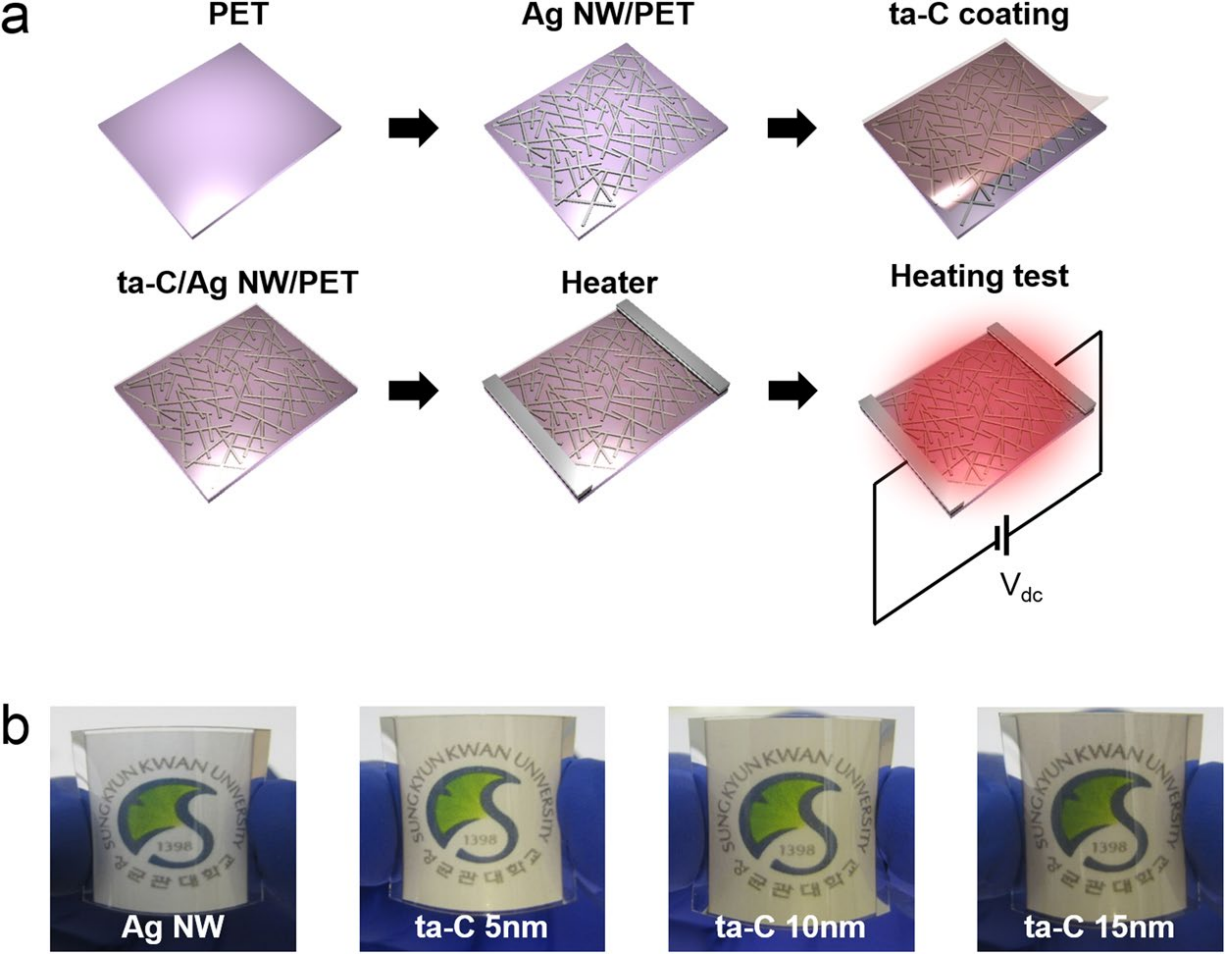
(**a**) Schematic fabrication process of the transparent and flexible TFHs on ta-C/Ag NW network electrode. Edge Ag contact electrodes effectively provide the external power into transparent electrode. (**b**) Pictures of curved TFHs on bare Ag NW and ta-C/Ag NW electrodes with different ta-C thickness.

To apply the ta-C coated Ag NW network as transparent electrode for TFHs, we fabricated the transparent and flexible TFHs on ta-C/Ag NW electrode with a size of 2.5 × 2.5 mm^2^ using a two-metal terminal side Ag contact configuration as shown is Fig. 6a. After preparation of ta-C coated Ag NW network electrode, the 100 nm thick Ag side metal contact was prepared by conventional DC sputtering by metals shadow mask. For heating of transparent and flexible TFHs, the DC voltage was directly supplied by power supply to the ta-C coated Ag NW electrode through DC sputtered Ag contact electrode at the film edge. Figure 6b show pictures of flexible and transparent TFHs fabricated on bare Ag NW and ta-C coated Ag NW electrode with increasing ta-C thickness. The curved TFHs demonstrated flexibility of ta-C coated Ag NW-based TFHs, which could be attached on curved surface. The temperature of TFHs was measured using a thermocouple directly mounted on the surfaces of TFHs and an IR thermal imager. In a black box, temperature of the transparent and flexible TFHs was measured with applying DC power with increasing voltage and time.

**Figure 7:**
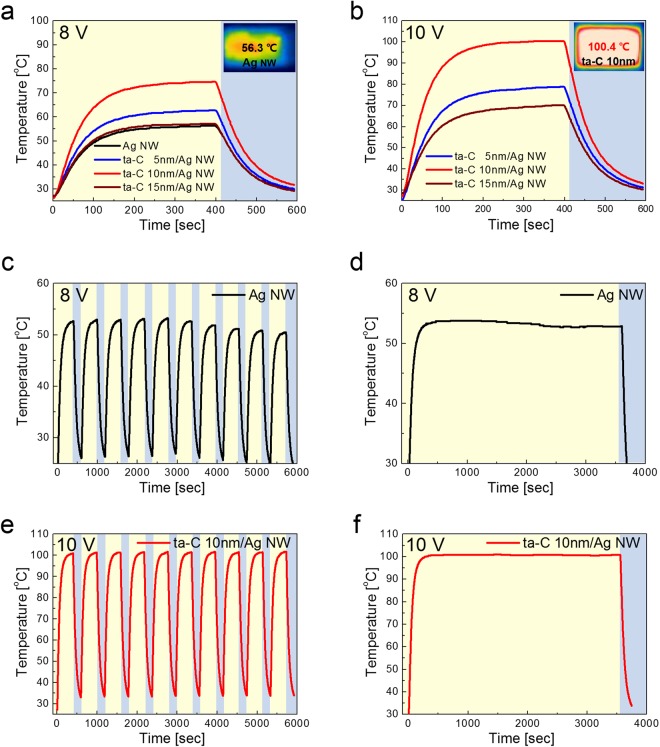
Temperature profiles of transparent and flexible and TFHs with bare Ag NW and ta-C/Ag NW electrodes at different DC input power of (**a**) 8 V and (**b**) 10 V. Inset IR image showed the thermal uniformity of TFH of bare Ag NW and ta-C/Ag NW electrode. (**c**) Repeated temperature of TFHs with bare Ag NW electrode and (**d**) temperature profile when a constant DC voltage of 8 V was supplied to the Ag NW electrode for 1 hour. (**e**) Repeated temperature profile of TFHs with ta-C (10 nm)/Ag NW electrode and (**f**) constant temperature profile when the DC voltage of 10 V was supplied to the ta-C (10 nm)/Ag NW electrode based TFHs for 1 hour.

Figure 7 shows temperature profiles of transparent and flexible TFHs on bare Ag NW and ta-C/Ag NW network electrode at different input DC power measured by thermocouples. Figure 7a is temperature profile of transparent and flexible TFHs fabricated on bare Ag NW and ta-C/Ag NW electrodes with different ta-C thickness at a constant input voltage of 8 V. When DC voltage was supplied to the Ag NW-based TFHs, the temperature of the all TFHs gradually increased and reached the saturation temperature. Compared to saturation temperature (56 °C) of bare Ag NW-based TFH, the TFH with ta-C/Ag NW electrode showed higher saturation temperature due to lower sheet resistance. At a constant input DC voltage of 8 V, the TFH with ta-C (10 nm)/Ag NW electrode showed highest saturation temperature of 74.6 °C due to lowest sheet resistance of 36.83 Ohm/square. However, further increase of input DC voltage up to 10 V led to failure of Ag NW-based TFH due to disconnection of Ag NWs during heating. Therefore, we can’t obtain the temperature profile of bare Ag NW based TFH at a input voltage of 10 V. The concentration of current on specific region of the Ag NW network led to melting and disconnection of Ag NW network and failure of Ag NW-based TFH. Figure 7b shows the temperature profiles of TFHs with the ta-C/Ag NW electrodes with different thickness of ta-C layer. The temperature of the TFHs with a ta-C/Ag NW electrode increased as shown in all of the temperature profiles at constant input DC voltage. When the DC voltage of 10 V was supplied to ta-C/Ag NW-based TFHs, the TFH with ta-C (10 nm)/Ag NW electrode reached at a saturation temperature 100.4 °C, which is enough to evaporate water droplets or remove frost on the surface of TFH. However, the TFHs with 5 nm and 15 nm thick ta-C passivation layer did not reached at a temperature of 100 °C. It was noteworthy that the TFHs with ta-C (10 nm)/Ag NW showed the highest saturation temperature among the ta-C/Ag NW based TFHs due to lowest sheet resistance. Based on Joule’s law, we can correlate the sheet resistance of ta-C/Ag NW electrode and the generated temperature of the TFHs. The main heat loss in TFHs could be attributed to conduction loss in the substrate, convection loss in air and radiation loss. In our TFHs samples, heat loss due to conduction was negligible because the sample was prepared on insulating PET substrate. In addition, heat loss due to radiation was negligible below 100 °C due to the very low emissivity of the materials. Therefore, air convection is the main path of heat dissipation in the ta-C (10 nm)/Ag NW electrode-based TFHs^5,57^.

1 $${\rm{Qconv}}=\frac{{{\rm{V}}}^{2}}{{\rm{R}}}{\rm{\Delta }}{\rm{t}}$$

2 $${{\rm{T}}}_{{\rm{S}}}=\frac{{{\rm{V}}}^{2}{\rm{\Delta }}{\rm{t}}}{{{\rm{Rh}}}_{{\rm{conv}}}{{\rm{A}}}_{{\rm{conv}}}}$$

In equations (1) and (2), Q_conv_ is heat loss by convection, R is resistance of electrode, Δt is operation time, h_conv_ is a convective heat transfer coefficient, A_conv_ is the surface area, and T_s_ and T_i_ are the saturation and initial temperature, respectively. Based on equation (2), it in apparent that the saturation temperature of TFHs increase with increasing input DC voltage (V) and with decreasing resistance (R). Therefore, a lower sheet resistance of a ta-C/Ag NW is closely related to the higher saturation temperature of TFHs. To investigate durability of TFHs with Ag NW and ta-C/Ag NW electrodes, we performed repeated heating-cooling test for 10 cycles and kept the saturating temperature for 1 hour. In case of TFH with bare Ag NW electrode, the saturation temperature gradually decreased with increasing heating-cooling cycling as shown in Fig. 7c. During heating of TFH, the surface of Ag NWs are easily sulfidized by reduction of H_2_S and resistance of Ag NW network increased. Due to increased sheet resistance, the TFH with bare Ag NW electrode showed gradually decreased saturation temperature. At constant DC voltage of 8 V, the saturation temperature also decreased with time due to resistance change of the Ag NW electrode as shown in Fig. 7d. Figure 7e shows the temperature profiles of the ta-C (10 nm)/Ag NW electrode-based TFHs for repeated 10 cycles. Unlike TFH with bare Ag NW electrode, TFHs with ta-C coated Ag NW electrode showed an identical temperature profiles and easily reached at a saturation temperature of 100 °C when DC voltage of 10 V was applied. In addition, when the DC voltage of 10 V was supplied to the ta-C (10 nm)/Ag NW electrode-based TFHs for 1 hour, the TFHs keep a saturation temperature of 100 °C steady without temperature modulation as shown in Fig. 7f. The durability of transparent and flexible TFH with ta-C/Ag NW electrode is attributed to the effective anticorrosion and passivation of ta-C layer on Ag NW network against sulfidation of Ag NWs. The conformal coating of ta-C layer on Ag NW effectively prevent the reduction of H_2_S with Ag NW. In addition, passivation of semiconducting ta-C layer led to bridge effect between Ag NWs and prevent the current concentration on specific Ag NWs. Therefore, combined effect of ta-C passivation improved the performance of transparent and flexible Ag NW-based TFHs. In addition, the low friction coefficient and high hardness of FCVA coated ta-C passivation layer improve the durability against external scratch or external impact^63^.

**Figure 8:**
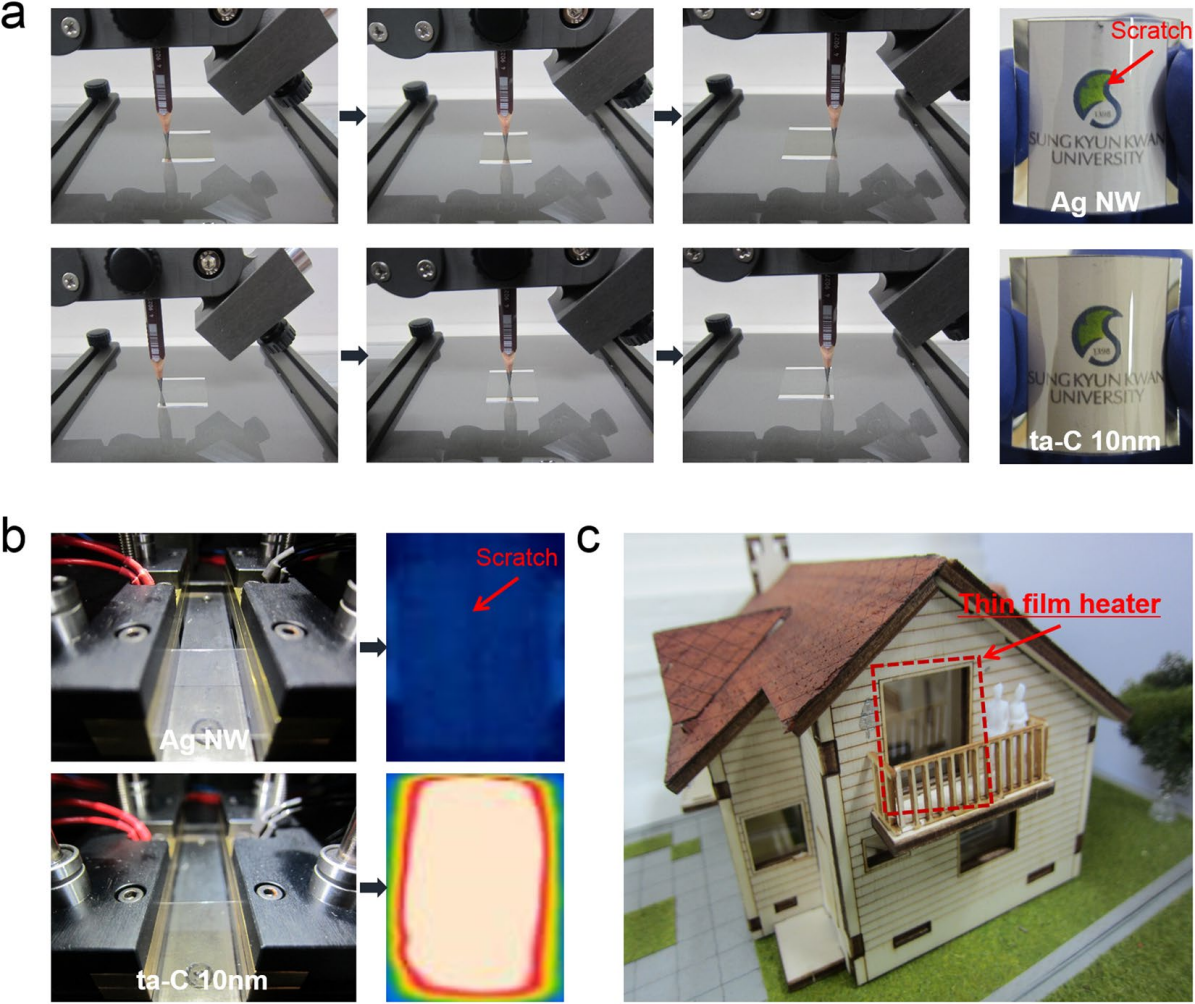
(**a**) Comparison of pencil hardness test of bare Ag NW and ta-C/Ag NW electrode for TFHs. Arrow indicates the scratch of the Ag NW after pencil test. (**b**) IR images of TFHs with Ag NW and ta-C/Ag NW electrode after pencil test. (**c**) Promising application of transparent TFHs in smart house.

Figure 8a showed pencil (6 H) hardness test of TFHs with bare Ag NW and ta-C/Ag NW electrodes. After pencil test, the TFH with bare Ag NW electrode showed scratch as indicated by arrow due to poor adhesion of Ag NWs. However, there is no scratch on the TFH with ta-C coated Ag NW after pencil hardness test due to durability and low fraction coefficient of ta-C passivation layer. Therefore, the TFH with bare Ag NW electrode was not work after pencil hardness teas as shown in Fig. 8b because the Ag NW electrode was completely disconnected by scratch. However, the TFH with ta-C/Ag NW electrode showed same performance even after pencil hardness test because the ta-C layer effectively prevent the scratch. Due to reliability and durability of TFHs with ta-C/Ag NW electrode, the transparent and flexible TFHs with ta-C/Ag NW could be used as smart window for automobiles and smart house. As shown in Fig. 8c, the smart window equipped with ta-C/Ag NW based transparent TFHs in smart house could easily remove the frost on the window during winter. Therefore, the ta-C/Ag NW hybrid film is promising transparent and flexible electrode for high performance TFHs due to its low sheet resistance, high optical transmittance, outstanding flexibility and hardness for external force.

## Discussion

We demonstrated that highly stable Ag NW electrode passivated by transparent ta-C layer for high performance transparent and flexible TFHs. The thickness effect of FCVA coated ta-C film on the electrical, optical, and mechanical properties of Ag NW electrode was investigated in detail to optimize the thickness of the ta-C layer. Due to bridge effect of FCVA coated ta-C film, the ta-C/Ag NW electrode showed lower sheet resistance than bare Ag NW electrode. At optimized thickness (10 nm) of ta-C passivation layer, the ta-C/Ag NW electrode showed a sheet resistance of 36.83 Ohm/square and optical transmittance 81.99%. In addition, we investigated the mechanical integrity of ta-C/Ag NW electrodes based on lab-made bending test system. In spite of existence of the ta-C passivation layer, the ta-C/Ag NW electrode showed outstanding mechanical flexibility due to improved adhesion of Ag NWs. In particular, the TFHs fabricated on ta-C/Ag NW exhibited better performance and stability than the TFHs on bare Ag NW electrode due to the effective current spreading through the ta-C layer and passivation/anticorrosion effect of the ta-C layer. Effective heat generation performance and durability of the TFH with ta-C/Ag NW electrode indicates that FCVA coated ta-C passivation is promising passivation layer in Ag NW electrode to obtain high performance Ag NW based transparent and flexible TFHs.

## Methods

### Slot-die coating of Ag NW percolating network electrodes

Uniformly coated Ag NW percolating network films were prepared on a 700 mm wide PET substrate at room temperature using pilot-scale roll-to-roll (RTR) slot die coater (DKT 2015-R1-SHU500) as reported in our previous work^64^. The RTR slot die coating system consisted of ink tanks, a slot die coating zone, UV treatment zone. Then Ag NW coated films were moved to the heating zone (120 °C) by means of unwinding and rewinding at a roller speed of 2 m/min. After coating the Ag NWs, an over-coating layer was coated on the Ag NW layers to protect their degradation.

### Coating of ta-C passivation layer by FCVA

The ta-C passivation layer was directly deposited on the slot-die coated Ag NW network electrode using single-cathode of FCVA system^65^. The vacuum arc source with a T-shaped filter is attached to the bottom side of the coating chamber. A magnetic solenoid is used in the FCVA system to separate charged carbon ions from neutral atoms and macro- or micro-particles. A carbon target of 55 mm diameter and 99.99% purity is mounted on the bottom right side of the chamber. Those macro-particles and neutral carbon could be filtered out by a T-shaped filter. Therefore, only carbon with controlled energy range ions will be deposited on flexible substrate. The magnetic coil current is fixed to 5 A. The substrate holder was placed horizontally on a sample carrier for high-speed coating at a working pressure of 1 × 10^−5^ Torr without intentional substrate heating. A duct bias of 20 V and a substrate bias of 500 V were applied during the deposition. The Ar gas flowed at a rate of 2 sccm for stable arc plasma generation. The thickness of ta-C passivation was controlled by deposition time from 40 and 170 s.

### Characterization of ta-C/Ag NW electrodes

Sheet resistance of the ta-C coated Ag NW network electrodes and bare Ag NW electrodes were measured using a four point probe as a function of ta-C thickness. The optical transmittance of the ta-C coated Ag NW was measured by using a UV/visible spectrometer in a wavelength region between 200 and 1200 nm. The surface morphology of ta-C passivation layer on the Ag NW was examined using field emission scanning electron microscopy (FESEM) with increasing ta-C thickness. The mechanical flexibility of ta-C coated Ag NW and bare Ag NW electrodes were compared by lab-made bending test results. The resistance change (ΔR) of the ta-C coated Ag NW electrode during inner and outer bending was measured with decreasing bending radius to decide critical bending radius. In addition, dynamic fatigue tests of the ta-C coated Ag NW electrode was carried out at a fixed bending radius of 3 mm and a frequency of 1 Hz for 10,000 cycle to show the mechanical stability of the ta-C coated Ag NW electrodes.

### Fabrication and evaluations of the TFHs

To investigate the feasibility of FCVA coated ta-C passivation layer, flexible and transparent TFHs were fabricated on ta-C/Ag NW and bare Ag NW electrodes with a size of 2.5 × 2.5 mm^2^. For effective power supply on TFHs, 100 nm thick Ag metal side contact were fabricated on the side of transparent electrode (ta-C/Ag NW and Ag NW) using DC magnetron sputtering at a constant DC power of 100 W applied to a 3-inch Ag target at a Ar flow rate of 20 sccm and a working pressure of 3 mTorr. To generate heat on the TFHs, DC voltage was supplied by a power supply (OPS 3010, ODA technologies) into the TFHs through an Ag contact electrode. The temperature of the transparent and flexible TFHs samples was measured by thermocouple as a function of time. After the temperature profile was obtained, an infrared (IR) image was obtained in order to demonstrate uniformity of the temperature of the TFHs using IR thermal imager (A35sc, FLIR).

## References

[1] Zilberberg K, Riedl T (2016). Metal-nanostructures – a modern and powerful platform to create transparent electrodes for thin-film photovoltaics. J. Mater. Chem. A..

[2] Langley D (2013). Flexible transparent conductive materials based on silver nanowire networks: a review. Nanotechnology.

[3] He W, Ye C (2015). Flexible transparent conductive films on the basis of Ag nanowires: design and application: a review. J. Mater. Sci. Technol..

[4] Sannicolo T (2016). Metallic nanowire-based transparent electrodes for next generation flexible devices: a review. Small.

[5] Seo K-W, Kim M-Y, Chang H-S, Kim H-K (2015). Self-assembled Ag nanoparticle network passivated by a nano-sized ZnO layer for transparent and flexible film heaters. AIP Adv..

[6] Kim D (2013). Transparent flexible heater based on hybrid of carbon nanotubes and silver nanowires. Carbon.

[7] Li J (2014). A flexible and transparent thin film heater based on a silver nanowire/heat-resistant polymer composite. Macromol. Mater. Eng..

[8] Xu F, Zhu Y (2012). Highly conductive and stretchable silver nanowire conductors. Adv. Mater..

[9] Lee J (2012). Very long Ag nanowire synthesis and its application in a highly transparent, conductive and flexible metal electrode touch panel. Nanoscale.

[10] Huang Q (2015). Highly flexible and transparent film heaters based on polyimide films embedded with silver nanowires. RSC Adv..

[11] Im H-G (2014). Flexible transparent conducting composite films using a monolithically embedded AgNW electrode with about robust performance stability. Nanoscale.

[12] Lee P (2012). Highly stretchable and highly conductive metal electrode by very long metal nanowire percolation network. Adv. Mater..

[13] Kim TY (2013). Uniformly interconnect silver-nanowire networks for transparent film heaters. Adv. Funct. Mater..

[14] dos Reis Benatto GA (2016). Roll-to-roll printed silver nanowires for increased stability of flexible ITO-free organic solar cell modules. Nanoscale.

[15] Seo JH (2017). Cold isostatic-pressured silver nanowire electrodes for flexible organic solar cells via room-temperature processes. Adv. Mater..

[16] Tokuno T (2011). Fabrication of silver nanowire transparent electrodes at room temperature. Nano Res..

[17] Choi DY, Kang HW, Sung HJ, Kim SS (2013). Annealing-free, flexible silver nanowire-polymer composite electrodes via a continuous two-step spray-coating method. Nanoscale.

[18] Madaria AR, Kumar A, Zhou C (2011). Large scale, highly conductive and pattered transparent films of silver nanowires on arbitrary substrates and their application in touch screens. Nanotechnology.

[19] Amjadi M, Pichitpajongkit A, Lee S, Ryu S, Park I (2014). Highly stretchable and sensitive strain sensor based on silver nanowire-elastomer nanocomposite. ACS Nano.

[20] Hu L, Kim HS, Lee JY, Peumans P, Cui Y (2010). Scalable coating and properties of transparent, flexible, silver nanowire electrodes. ACS Nano.

[21] Elechiguerra JL (2005). Corrosion at the nanoscale: the case of silver nanowires and nanoparticles. Chem. Mater..

[22] Lee D (2013). Highly stable and flexible silver nanowire-graphene hybrid transparent conducting electrodes for emerging optoelectronic devices. Nanoscale.

[23] Seo K-W, Lee J-H, Kim H-J, Kim H-K, Na S-I (2014). Highly transparent and flexible InTiO/Ag nanowire/InTiO films for flexible organic solar cells. Appl. Phys. Lett..

[24] Kim D-H, Ko E-H, Kim K-H, Kim T-W, Kim H-K (2016). Transparent and flexible Ag nanowire network covered by a thin ITO layer for flexible organic light emitting diodes. ECS J. Solid State Sci. Technol..

[25] Zhang X, Yan X, Chen J, Zhao J (2014). Large-size graphene microsheets as a protective layer for transparent conductive silver nanowire film heaters. Carbon.

[26] Lee D, Lee H, Ahn Y, Lee Y (2015). High-performance flexible transparent conductive film based on graphene/AgNW/graphene sandwich structure. Carbon.

[27] Zhang W (2017). Highly conductive and transparent silver grid/metal oxide hybrid electrodes for low-temperature planar perovskite solar cells. J. Power Sources.

[28] Duan Y-H (2015). High-performance flexible Ag nanowire electrode with low-temperature atomic-layer-deposition fabrication of conductive-bridging ZnO film. Nanoscale Res. Lett..

[29] Yoo JH (2015). Silver nanowire-conducting polymer-ITO hybrids for flexible and transparent conductive electrodes with excellent durability. ACS Appl. Mater. Interfaces.

[30] Seo K-W (2014). Simple brush painted Ag nanowire network on graphene sheets for flexible organic solar cells. J. Vac. Sci. Technol. A.

[31] Robertson J (2002). Diamond-like amorphous carbon. Mater. Sci. Eng R.

[32] McKenzie DR (1996). Tetrahedral bonding in amorphous carbon. Rep. Prog. Phys..

[33] Lifshitz Y (2003). Pitfalls in amorphous carbon studies. Diamond Relat. Mater..

[34] Lifshitz Y (1999). Diamond-like carbon – present status. Diamond Relat. Mater..

[35] Neuville S, Matthews A (2007). A perspective on the optimization of hard carbon and related coating for engineering applications. Thin Solid Films.

[36] Klein F (2016). Nanoscale scanning electron microscopy based graphitization in tetrahedral amorphous carbon thin films. Carbon.

[37] Espinosa HD (2006). Elasticity, strength, and toughness of single crystal silicon carbide, ultrananocrystalline diamond, and hydrogen-free tetrahedral amorphous carbon. Appl. Phys. Lett..

[38] Ferrari AC (1999). Elastic constants of tetrahedral amorphous carbon films by surface brillouin scattering. Appl. Phys. Lett..

[39] Balandin AA (2008). Thermal conductivity of ultrathin tetrahedral amorphous carbon films. Appl. Phys. Lett..

[40] Palomäki T (2017). Electron transport determines the electrochemical properties of tetrahedral amorphous carbon (ta-C) thin films. Electrochim. Acta.

[41] Caro MA, Zoubkoff R, Acevedo OL, Laurila T (2014). Atomic and electronic structure of tetrahedral amorphous carbon surfaces from density functional theory: properties and simulation strategies. Carbon.

[42] Soin N (2012). Thickness dependent electronic structure of ultra-thin tetrahedral amorphous carbon (ta-C) films. Thin Solid Films.

[43] Bewilogua K, Hofmann D (2014). History of diamond-like carbon films – from first experiments to worldwide applications. Surf. Coat. Technol..

[44] Panwar OS (2013). Few layer graphene synthesized by filtered cathodic vacuum arc technique. J. Vac. Sci. Technol. B.

[45] Panwar OS (2006). Reflectance and photoluminescence spectra of as grown and hydrogen and nitrogen incorporated tetrahedral amorphous carbon films deposited using an S bend filtered cathodic vacuum arc process. Thin Solid Films.

[46] Panwar OS (2010). Effect of high substrate bias and hydrogen and nitrogen incorporation on spectroscopic ellipsometric and atomic force microscopic studies of tetrahedral amorphous carbon films. Surf. Coat. Technol..

[47] Kesarwani AK (2016). Determining the number of layers in graphene films synthesized by filtered cathodic vacuum arc technique. Fullerenes Nanotubes Carbon Nanostruct..

[48] Panwar OS (2008). Effect of hydrogen and nitrogen incorporation on the properties of tetrahedral amorphous carbon films grown using S bend filtered cathodic vacuum arc process. Indian J. Pure Appl. Phys..

[49] Panwar OS (2009). Characterization of boron-and phosphorous-incorporated tetrahedral amorphous carbon films deposited by the filtered cathodic vacuum arc process. Jpn. J. Appl. Phys..

[50] Panwar OS (2004). Space charge limited conduction and electron paramagnetic resonance studies of as grown and nitrogen incorporated tetrahedral amorphous carbon films deposited by pulsed unfiltered cathodic vacuum arc process. Diamond Relat. Mater..

[51] Grierson DS (2010). Thermal stability and rehybridization of carbon bonding in tetrahedral amorphous carbon. J. Appl. Phys..

[52] Yang X, Haubold L, Devivo G, Swain GM (2012). Electroanalytical performance of nitrogen-containing tetrahedral amorphous carbon thin-film electrodes. Anal. Chem..

[53] Liu A (2010). Non-enzymatic hydrogen peroxide detection using gold nanoclusters-modified phosphorus incorporated tetrahedral amorphous carbon electrodes. Electrochim. Acta.

[54] Ferrari AC, Kleinsorge B, Morrison NA, Robertson AHS (1999). Stress reduction and bond stability during thermal annealing of tetrahedral amorphous carbon. J. Appl. Phys..

[55] Jang Y-J, Kang Y-J, Kitazume K, Umehara N, Kim J (2016). Mechanical and electrical properties of micron-thick nitrogen-doped tetrahedral amorphous carbon coatings. Diamond Relat. Mater..

[56] Chen BJ, Sun XW, Divayana Y, Tay BK (2005). Improving organic light-emitting devices by modifying indium tin oxide anode with an ultrathin tetrahedral amorphous carbon film. J. Appl. Phys..

[57] Kim D-H, Cho K-S, Kim H-K (2017). Thermally evaporated indium-free, transparent, flexible SnO_2_/AgPdCu/SnO_2_ electrodes for flexible and transparent thin film heaters. Sci. Rep..

[58] Kang S-B, Noh Y-J, Na S-I, Kim H-K (2014). Brush-painted flexible organic solar cells using highly transparent and flexible Ag nanowire network electrodes. Sol. Energy Mater. Sol. Cells.

[59] Song C-H (2015). Intense-pulsed-light irradiation of Ag nanowire-based transparent electrodes for use in flexible organic light emitting diodes. Org. Electron..

[60] Liu C-H, Yu X (2011). Silver nanowire-based transparent, flexible, and conductive thin film. Nanoscale Res. Lett..

[61] Park J-Y, Hong S, Jang J, Kim H-K (2017). Blue laser annealing of Ag nanowire electrodes for flexible thin film heaters. ECS J. Solid State Sci. Technol..

[62] Lim J-E (2017). Brush-paintable and highly stretchable Ag nanowire and PEDOT:PSS hybrid electrodes. Sci. Rep..

[63] Panwar OS, Khan MA, Satyanarayana BS, Kumar S (2010). & Ishpal. Properties of boron and phosphorous incorporated tetrahedral amorphous carbon films grown using filtered cathodic vacuum arc process. Appl. Surf. Sci..

[64] Kim D-J (2016). Roll-to-roll slot-die coating of 400 mm wide, flexible, transparent Ag nanowire films for flexible touch screen panels. Sci. Rep..

[65] Jang Y-J, Kim GT, Kang Y-J, Kim DS, Kim J-K (2016). A study on thick coating of tetrahedral amorphous carbon deposited by filtered cathode vacuum arc plasma. J. Mater. Res..

